# Comparison of the clinical effectiveness of self-etch sealant with the conventional acid-etch sealant for the retentive ability and marginal integrity in permanent molars: a split mouth one year randomized controlled trial

**DOI:** 10.12688/f1000research.109584.1

**Published:** 2022-03-02

**Authors:** Deveshi Gupta, Arathi Rao, Ramya Shenoy, Baranya Srikrishna Suprabha

**Affiliations:** 1 Pediatric & Preventive Dentistry, Manipal College of Dental Sciences, Mangalore, Manipal Academy of Higher Education, Manipal, Karnataka, 575001, India; 2 Public Health Dentistry, Manipal College of Dental Sciences, Mangalore, Manipal Academy of Higher Education, Manipal, Karnataka, 575001, India

**Keywords:** Self-etch, Acid etch, Sealants, Retention, pit and fissure, marginal integrity, discoloration, ClinPro, Preventseal

## Abstract

**Background: **Self-etching has been shown to be beneficial compared to the other resin sealants especially in pediatric practice. The present *in-vivo* study was designed to clinically evaluate the sealing ability and retention of the self-etching sealant compared to the conventional resin sealant. The aim was to evaluate and compare the retention and marginal integrity of the self-etch and acid etch sealant materials at three, six and twelve-month follow up.

**Methods: **This was designed a randomized controlled trial, with double blind parallel, split-mouth design. In total, 35 children (70 teeth) between 7 and 10 years of age formed part of the study. Group 1 received acid-etch sealant and Group 2 received self-etch sealants. The study participants and the investigator who performed the statistical analysis were blinded to the treatment allocation. All the samples were evaluated at 3, 6, and 12 months. The inter-group and intragroup comparison were carried out using the Chi-Square test and Friedman test with level of significance set at 5% and the P value less than 0.05.

**Results: **Complete retention of sealants was observed in 34.5% of conventional acid etch (group 1) and 22.9% of self-etch samples (group 2) whereas complete loss of sealants were seen in 11.4% of group 1 and 20% of the group 2 samples and intergroup comparison of retention failure was non-significant (p=0.135). In total, 85.7% of the group 1 and 82.9% of the group 2 samples exhibited good marginal integrity with no clinical changes in the enamel around the margins but this was not statistically significant (p=0.5).

**Conclusions: **Sealants with fewer procedural steps and those which provide adequate retention would be ideal for use in children. Thus, self-etch sealants have been found to be effective and efficient as a sealant in the present *in-vivo* study.

**Clinical Trials Registry, India registration:** CTRI/2019/03/018343 (29/03/2019).

## Introduction

Occlusal caries still continue to be the most common form of decay in the permanent dentition due to the presence of deep retentive pits and fissures. Their prevention is a challenge that can be controlled by non-operative measures especially if implemented early. ^1^^,^^2^ American Academy of Pediatric Dentistry and Cochrane recommend the application of pit-and-fissure sealants on newly erupted posterior teeth so as to reduce the incidence of pit and fissure caries. ^3^^,^^4^ Sealant retention is crucial for it to be effective, and this can be achieved by various factors. ^5^

Etching the enamel with phosphoric acid is a conventional and standard technique practiced globally to improve retention. ^6^ Unfortunately, salivary contamination post etching, decreases the adhesion of the sealant due to the formation of a tenacious surface coating, thus requiring the etching procedure to be repeated. Considering the compliance of the young child, a sealant that provides good retention and having a short procedure of application would be ideal. ^6^

Self-etching sealants are products that have a one-step application with fluoride releasing properties. They can prove to be beneficial compared to the other resin sealants especially in pediatric practices. ^2^ There have been many *in-vitro* evaluation studies ^7^^,^^8^ conducted comparing the self-etch sealant with conventional acid etch sealants. The present *in-vivo* study was thus designed with the focus to clinically evaluate the sealing ability and retention of the self-etching sealant compared to the conventional resin sealant.

### Objective and hypothesis

The objective of this study was to evaluate and compare the retention of the sealant material and marginal enamel changes at three, six and 12-month follow up following sealant placement. The null hypothesis was that there would be no difference in the retention and marginal enamel structure between the self-etch and conventional acid-etch sealant at three, six and 12-month follow up.

## Methods

### Study settings

The study was conducted in the Department of Paediatric and Preventive Dentistry, Mangalore, South India and was a self-funded study.

### Study design

This study was a randomized controlled trial, with a double blind parallel, split-mouth study design and 1:1 allocation ratio. The investigation was designed according to the Consolidated Standards of Reporting Trials (CONSORT). Completed CONSORT and TIDieR checklists can be found in the *Reporting guidelines* section. ^22^^–^^24^

### Ethical approval

All procedures were performed in conformity with the ethical standards of the institutional research committee and the Helsinki declaration and its amendments. Ethical clearance was obtained from the Institutional Ethics Committee, Manipal College of Dental Sciences, Mangalore prior to the study (Protocol no: 19025, dated 18 ^th^ February 2019). The trial was registered at the Clinical Trials Registry, India with submission number as CTRI/2019/03/018343 on 22 ^nd^ November 2019.

### Informed consent

Written informed consent was obtained from the parents and written informed assent was obtained from the individual participants of the study. It was ensured that participation in the study was entirely based on the will of the participants and their parents. They were also assured that their decision to participate or not would not affect the dental treatment services for the child. The participants were free to withdraw from the study at any time. Participation was voluntary and no compensation or incentives were provided to the participants. The information sheet and consent form can be found as *Extended data.* ^21^

### Sample size

Sample size was calculated at 80% power and 90% confidence interval with clinically acceptable margin and proportion of retention of sealants as 50% in each group. Compensating for the loss to follow up at 10%, the minimum final sample size was calculated as 64 teeth, 32 in each group.

### Eligibility criteria

Over a span of six-months, 50 children between the ages of 7-10 years, who visited the department for routine dental check-up were examined during their initial clinical examination. Mouth mirrors and probes were used to examine the children’s teeth under good chairside illumination. After screening, the children with fully erupted first permanent molars having deep pit and fissures without any signs of caries on any surface of the same teeth were selected. There was also a need that there should be two teeth that fulfilled the criteria one on each side of the arch. In addition, in order to be included into the study, each child should have presented with Decay, Missing, Filled Surface (DMFS) index of ≥1 ^9^ in the form of visible cavities, fillings or missing teeth due to caries, with a co-operative behaviour (Frankl score 3 or 4) ^10^ and satisfactory oral hygiene (OHI-S ≤ 3). ^11^ Children with a history of any medical conditions or long-term medications that affected the salivary flow were not included. Additionally, children with pernicious oral habits, undergoing orthodontic treatment or wearing intraoral devices, permanent molars with developmental defects or restorations and/or history of allergies to resins were also excluded.

### Method of randomization

A coin toss was used to allocate the tooth to each group. Randomization was carried out by an operator who was not involved in any other phases of the clinical trial. The unit of randomization was the individual tooth. A coin was tossed to determine which tooth the conventional acid etch pit and fissure sealant (Group 1: 3M™ ClinPro™, India, 3M ID 70201412429) ^12^ would be applied to. If the coin landed as Heads, the sealant was placed on the right mandibular first permanent molar. Subsequently, the self-etch pit and fissure sealant (Group 2: Preventseal (Itena ^®^, France, PVSEAL-1.2) ^13^ was applied on the left mandibular molar. Similarly, if the coin landed as Tails, the conventional sealant was placed on the left mandibular first permanent molar and the self-etch pit and fissure sealant was applied on the right mandibular molar.

The group allocation was revealed to the principal investigator who performed the intervention just before the sealant placement procedure began by the operator who had carried out the randomization.

### Blinding

The study participants and the investigator who performed the statistical analysis were blinded to the treatment allocation. However, the operator (DG) who performed the intervention and the examiner (AR) who evaluated the teeth were not blinded due to the difference in the color of the materials. ClinPro™ Sealant is initially pink and changes to white following curing, while Preventseal is cream white material. A single examiner (AR) evaluated all the teeth at the period of 3, 6 and 12 months.

### Intervention

The trial commenced from March 2019 to April 2020 and all the follow-up examinations were completed by 15 ^th^ April 2021. Both the study materials were purchased by the investigators and were used within their labeled shelf life.

### Procedure

The selected teeth were cleaned with an ultrasonic scaler and then polished with a non-fluoridated prophylactic paste using slow speed hand piece and rubber cup. The teeth were adequately rinsed with water and then air-dried. Cotton rolls and saliva ejectors were used for isolation during the procedure.

The sealants were applied by a qualified dental surgeon in accordance with the manufacturer’s instructions, on the occlusal pit and fissures of the first permanent molars.

**Group 1 (**3M™ ClinPro™, Conventional acid etch sealants **):** The entire fissure was etched for 30 seconds with 37% phosphoric acid gel using an applicator tip. The teeth were then rinsed using water for 20 seconds and dried with oil free compressed air. A conventional fissure sealant was applied as per the manufacturers’ instructions. A probe was used to remove any air bubbles and the margins were thoroughly checked. The sealant was then polymerized using a visible light curing unit for 30 seconds.

**Group 2 (**Preventseal (Itena ^®^, Self-etch sealants): The self-etching sealant was applied to the tooth and allowed to penetrate the fissures for 20 seconds, as per the manufacturers’ instructions. A probe was used to remove any air bubbles and the margins were thoroughly checked. The sealant was then polymerized using a visible light curing unit for 30 seconds.

The occlusion was verified with an articulating paper and the high points if any, were subsequently reduced using a slow speed finishing bur. The finished sealants were checked for voids using a probe, which if present were refilled. All the sealant applications were crosschecked using a mouth mirror and probe by an experienced examiner. The light intensity output was maintained at 600 mW/cm ^2^ with the help of a radiometer (Demetron 100, Demetron Research Corp, Danbury, CT, USA). The light output was checked after every two patients.

### Outcomes

1. Sealant retention

The samples in both the group were evaluated at 3, 6, and 12 months from the date of treatment. All examinations were carried out using the visual and tactile method, with the help of mouth mirror and probe under good chair side illumination by a single examiner.

Sealant retention was recorded according to Simonsen’s criteria ^14^ as follows: Score 0: Complete loss of sealant; Score 1: Sealant fully retained; Score 2: Partial loss of sealant.

2. *Marginal integrity* ^15^

Enamel at the sealant margins were closely checked and evaluated. An alteration in colour, loss of translucency and defects along the margins were considered as loss of marginal integrity and given a score of 1. Concurrently, the teeth with no such changes were given a score of 2 and considered as intact marginal integrity.

### Statistical analysis

All the data was entered and analyzed using Statistical Package for Social Science ( SPSS), version 17 (SPSS Inc. Chicago, IL, USA, RRID:SCR_019096) at the completion of the 12-month follow up examination. The frequency distribution for fully-retained, partially retained and complete loss of retention was tabulated. Similarly, the percentage distribution for presence and absence of marginal changes was calculated. The inter-group and intragroup comparison of sealant retention and marginal discoloration at 3 months, 6 months, and 12 months was carried out using the Chi-Square test and Friedman test. For all the tests, the level of significance was set at 5%. The P value was kept less than 0.05.

## Results

**Figure 1.**
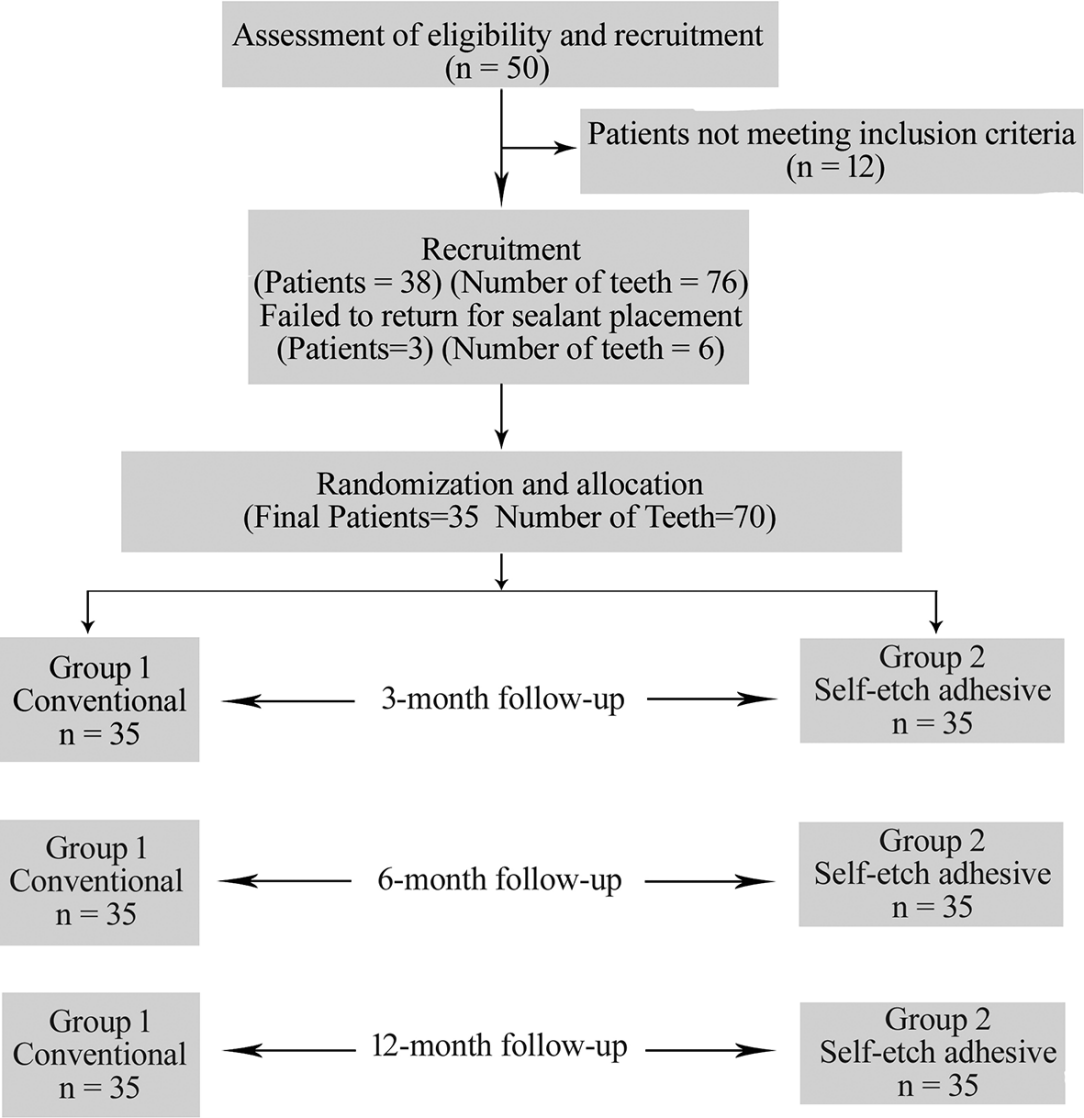
CONSORT flow chart.

Out of the initial 50 children, 38 participants were selected (76 teeth) and were recruited. ^20^ Out of them 3 children failed to report for sealant placement and did not respond to our calls as depicted in the flow chart ( Figure 1).

The final study samples consisted of 15 males and 20 females with the mean age of 8.5 years.

Teeth with partial/complete loss of sealants were repaired and were then subsequently dropped from the study. Their scores, however, were carried forward into the successive follow up appointments.

Complete retention of sealants was observed in 34.5% of group 1 and 22.9% of group 2 samples, whereas complete loss of sealants was seen in 11.4% of group 1 and 20% of the group 2 samples. Partial loss of sealants was more common in both the groups than complete loss. Group 1 had more teeth (34.3%) with complete retention than group 2 (22.9%) at the end of 12 months. Most of the retention failure was seen in the first evaluation period. Intergroup comparison of Simonsen’s Score for sealant retention between group 1 and 2 at follow-up was done using chi-square test and was found to be non-significant (p=0.135) ( Table 1).

**Table 1.** Intergroup comparison of Simonsen’s Score for sealant retention between ClinPro™ Sealant and Preventseal at 3, 6, and 12 months.

Group N=35 each group	Retention score	3 months n(%)	6 months n(%)	12 months n(%)	Chi-Square DF p Value	Chi-Square DF p Value
**Group 1 ClinPro™ Sealant**	0	4(11.4)	4(11.4)	4(11.4)	**2.000** **2** **0.368**	**4.00** **2** **0.135**
	1	13(37.2)	13(37.2)	12(34.3)		
	2	18(51.4)	18(51.4)	19(54.3)		
**Group 2 Preventseal**	0	7(20.0)	7(20.0)	7(20.0)	**2.000** **2** **0.368**	
	1	9(25.7)	9(26.5)	8(22.9)		
	2	19(54.3)	19(54.3)	20(57.1)		
**Chi-Square** **DF** **p Value**		**1.57** **2** **0.456**	**1.57** **2** **0.456**	**1.64** **2** **0.440**		

Score 0: Complete loss of sealant, Score 1: Sealant fully retained, Score 2: Partial loss of sealant.

DF: Degrees of freedom; P: Probability.

Overall, 85.7% of the group 1 and 82.9% of the group 2 samples exhibited good marginal integrity with no clinical changes in the enamel around the margins. Few of the teeth in both the groups exhibited discoloration and roughness but this was not statistically significant (p=0.5). In both the groups all the failures were seen by the first evaluation period at 3 months ( Table 2).

**Table 2.** Intergroup comparison of the marginal integrity at 3, 6, and 12 months.

	Marginal integrity score	3 months n(%)	6 months n(%)	12 months n(%)
**Group 1 ClinPro™ Sealant**	1	5(14.3)	5(14.3)	5(14.3)
	2	30(85.7)	30(85.7)	30(85.7)
**Group 2 Preventseal**	1	6(17.1)	6(17.1)	6(17.1)
	2	29(82.9)	29(82.9)	29(82.9)
**Chi-Square** **DF** **p Value**		**0.108** **1** **0.500**	**0.108** **1** **0.500**	**0.108** **1** **0.500**

Score 1: An alteration in colour, loss of enamel translucency and defects along the enamel margins.

Score 2: No changes along the enamel margin.

DF: Degrees of freedom; P: Probability.

## Discussion

In the earlier studies using different products, those sealants that had been applied using self-etch systems had shown lower retention rates than the sealants applied in the conventional approach, regardless of the use of adhesive systems. ^14^^,^^15^ In an *in vitro* study by Garg et al, ^6^ the sealing ability and microleakage of Preventseal and Clinpro™ were evaluated. They documented that the retention and marginal integrity of the self-etching sealant, Preventseal was found to be similar to that of the conventional acid etch sealant.

Based on the above information, we designed our *in-vivo* study to clinically compare the retention and marginal integrity between Preventseal and Clinpro™ sealants. Clinpro™ Sealant is a light-cured, fluoride releasing pit and fissure sealant with a color-changing feature. It is pink when applied to the tooth surface, and changes to an opaque off-white color when exposed to light. It contains 2, 2-bis[4-(2-hydroxy-3 methacryloxypropoxy)phenyl] propane, tri (ethylene glycol) dimethacrylate, a light cure initiator system based on camphorquinone, a tertiary amine and an iodonium salt. ^12^ Preventseal is a self-etched, light cured, fluoride releasing pit and fissure sealant. As the name suggests it requires no etching, rinsing or drying. It contains Bis-GMA, and triethyleneglycoldimethacrylate - 2-Hydroxyethylmethacrylate. ^13^

A split mouth design was used to reduce the inter-subject variability and ensure that each of the sealants did not influence the outcome of the other segments. It also reduced the sample size and improved our statistical efficiency as each patient served as his/her own control and created a relatively smooth follow-up. Unfortunately, each design has its own merits and demerits. The attrition of even one patient in this trial amounted to loss of data in all the fields and was hence considered when the sample size was calculated. ^16^ Further, this study was double blinded as both the data analyst and the participants were unaware of the type of sealant applied. The coin toss method of randomisation ensured that the children’s teeth were equally allocated to both groups of sealants. ^17^

This study’s inclusion criteria were drafted keeping the recommendations from AAPD and the British Society of Pediatric Dentistry in mind. ^3^^,^^18^ Based on these guidelines, sealants should be applied to the child’s first permanent molar between the ages of 7–10 years. ^17^ This ensures that the newly erupted teeth are protected from demineralization. Additionally, only children with fully erupted first permanent molars were included, to reduce the risk of bias caused by moisture contamination during sealant application. ^19^

All scientific measurements come with some degree of imprecision, hence the criteria was selected that minimized this to possible extent.

Although Clinpro™ exhibited better retention, the results were non-significant. Thus, it can be concluded that the self-etch sealant, Preventseal is as effective as Clinpro™ Sealant with adequate retention and marginal integrity and the null hypothesis is accepted.

A potential trial limitation is that since the two materials have different color, the operator and the clinical evaluator could not be blinded, which could have led to potential bias. To minimize this, the data analyst was blinded to the group allocation.

## Conclusion

Sealants with less procedural steps and those that provides adequate retention are ideal to be used in children. Thus, Preventseal has proved to be effective and efficient as a sealant with its self-etch and fluoride releasing ability even in the present *in-vivo* study.

## Data availability

### Underlying data

Figshare: unidentifiable Data. https://doi.org/10.6084/m9.figshare.19207596. ^20^

### Extended data

Figshare: Information sheet, consent form, accent form. https://doi.org/10.6084/m9.figshare.19196297. ^21^

### Reporting guidelines

Fighsare: CONSORT checklist and flow diagram for ‘Comparison of the clinical effectiveness of self-etch sealant with the conventional acid-etch sealant for the retentive ability and marginal integrity in permanent molars: a split mouth one year randomized controlled trial’.

https://doi.org/10.6084/m9.figshare.19130072 ^22^ & https://doi.org/10.6084/m9.figshare.19130081. ^23^ TIDieR Checklist. https://doi.org/10.6084/m9.figshare.19130075. ^24^

Data are available under the terms of the Creative Commons Attribution 4.0 International license (CC-BY 4.0).
