# Supplementary material for: Comparison of  clinical effectiveness of conventional and self-etch sealant: a split mouth randomized controlled trial
Source: F1000Res. 2022 Apr 19;11:261. Originally published 2022 Mar 2. [Version 3] doi: 10.12688/f1000research.109584.3 (PMC9044499; doi:10.12688/f1000research.109584.3)
Supplement: Peer review history

# Peer review: Reviewer response for version 3

**DOI:** 10.5256/f1000research.129991.r135132

**Published:** 2022-04-25

**Authors:** Muthu Murugan

**Affiliations:** 1 Centre for Early Childhood Caries Research (CECCRe), Department of Pediatric and Preventive Dentistry, Sri Ramachandra Faculty of Dental Sciences, Sri Ramachandra Institute of Higher Education and Research, Chennai, Tamil Nadu, India

All corrections are executed. Queries answered.

Is the work clearly and accurately presented and does it cite the current literature?

Yes

If applicable, is the statistical analysis and its interpretation appropriate?

Partly

Are all the source data underlying the results available to ensure full reproducibility?

Yes

Is the study design appropriate and is the work technically sound?

Yes

Are the conclusions drawn adequately supported by the results?

Yes

Are sufficient details of methods and analysis provided to allow replication by others?

Partly

Reviewer Expertise:

Early Childhood Caries

I confirm that I have read this submission and believe that I have an appropriate level of expertise to confirm that it is of an acceptable scientific standard.

# Peer review: Reviewer response for version 2

**DOI:** 10.5256/f1000research.122485.r127880

**Published:** 2022-04-12

**Authors:** Muthu Murugan

**Affiliations:** 1 Centre for Early Childhood Caries Research (CECCRe), Department of Pediatric and Preventive Dentistry, Sri Ramachandra Faculty of Dental Sciences, Sri Ramachandra Institute of Higher Education and Research, Chennai, Tamil Nadu, India

Majority of the corrections or suggestions are executed. Find my comments below:

**In the Introduction**

**The last paragraph of the Introduction the objective can be rephrased**

 from:

*The objective of this study was to evaluate and compare the retention of the sealant material and marginal enamel changes at three, six and 12-month follow up following sealant placement.*

 to:

*'The objective of this study was to evaluate the retention of the sealant material and marginal enamel changes at 3, 6 and 12-months following sealant placement.'*

**In the methods section**

**Blinding**

**The following lines from blinding to be moved under the heading ethical approval.**

*The trial commenced from March 2019 to April 2020 and all the follow-up examinations were completed by 15 ^th^ April 2021.*

*Also the Trial registered date is in Nov 2019. The trial started before the registration? Pls clarify. or There any errors in the study starting date. Pls edit accordingly.*

**The following line can be removed from blinding and placed in the intervention section.**

*Both the study materials were purchased by the investigators and were used within their labelled shelf life.*

**Results**

 The following lines in the results sections can be rephrased (language needs to be edited)

***Out of the initial 50 children,** 38 participants were selected (76 teeth) and were recruited. ^20^ **Out of them 3 children** failed to report for sealant placement and did not respond to our calls as depicted in the flow chart ( Figure 1).*

Is the work clearly and accurately presented and does it cite the current literature?

Yes

If applicable, is the statistical analysis and its interpretation appropriate?

Partly

Are all the source data underlying the results available to ensure full reproducibility?

Yes

Is the study design appropriate and is the work technically sound?

Yes

Are the conclusions drawn adequately supported by the results?

Yes

Are sufficient details of methods and analysis provided to allow replication by others?

Partly

Reviewer Expertise:

Early Childhood Caries

I confirm that I have read this submission and believe that I have an appropriate level of expertise to confirm that it is of an acceptable scientific standard, however I have significant reservations, as outlined above.

# Peer review: Reviewer response for version 1

**DOI:** 10.5256/f1000research.121099.r126008

**Published:** 2022-03-14

**Authors:** Muthu Murugan

**Affiliations:** 1 Centre for Early Childhood Caries Research (CECCRe), Department of Pediatric and Preventive Dentistry, Sri Ramachandra Faculty of Dental Sciences, Sri Ramachandra Institute of Higher Education and Research, Chennai, Tamil Nadu, India

This article evaluates the clinical performance of self etch versus conventional acid etch sealant by a Split mouth RCT. 

**Abstract:**

- It has few typos such as - First line in Methods - replace "a randomized controlled trial" with " as a randomized controlled trial" or " A double blinded, split mouth randomized controlled trial was carried out  

**Title:**

- Can be altered to - Comparison of Clinical Effectiveness of Conventional and Self-etch Sealant - A split mouth Randomized Controlled Trial.

**Introduction:**

- "The need for this research could have been emphasized in the introduction section. 
- The heading Objective and Hypothesis to be removed and the last paragraph to be merged with Introduction. 

**Methods:**

- Design cannot be both - Parallel and Split mouth. 
- Randomization - Too many repetitions. Was any allocation concealment was done? 
- Page 4 - Intervention - the statements ‘ The trial commenced from March 2019 to April 2020 and all the follow-up examinations were completed by 15th April 2021. Both the study materials were purchased by the investigators and were used within their labeled shelf life’ cannot be the intervention, please check and rephrase. Page 6, third line -Please mention after how many months in the statement - ‘Complete retention of sealants was observed in 34.5% of group 1 and 22.9% of group 2 samples, whereas complete loss of sealants was seen in 11.4% of group 1 and 20% of the group 2 samples’

**Discussion:**

- It needs to be re-structured.
- The results need to be compared with previous studies.
- Please mention limitations about carrying out toss coin randomization.
- Please highlight the study limitations of using a split mouth study design - First, the split-mouth design may lead to biased treatment efficacy estimates due to carry-across effects which (most likely) will be low or absent. However, there is even no statistical test that can detect this carry-across effect. Second, bias in recruitment of patients because of the need for symmetrical disease patterns among all segments of the dentition that are randomized and this limits the external validity of the results. Please refer to Lesaffre *et al. *(2009 ^1^) for more details.

**Conclusion:**

- Fluoride releasing ability of Preventseal wasn’t assessed in the present study hence in my opinion it shouldn’t be included here.

**Language:**

- There is room for improvement. Professional language edit of the entire manuscript can be helpful. Either American or British English to be used throughout.

Is the work clearly and accurately presented and does it cite the current literature?

Yes

If applicable, is the statistical analysis and its interpretation appropriate?

Partly

Are all the source data underlying the results available to ensure full reproducibility?

Yes

Is the study design appropriate and is the work technically sound?

Yes

Are the conclusions drawn adequately supported by the results?

Yes

Are sufficient details of methods and analysis provided to allow replication by others?

Partly

Reviewer Expertise:

Early Childhood Caries

I confirm that I have read this submission and believe that I have an appropriate level of expertise to confirm that it is of an acceptable scientific standard, however I have significant reservations, as outlined above.

## References

[1] : The design and analysis of split-mouth studies: what statisticians and clinicians should know. *Stat Med* .2009;28(28) : 10.1002/sim.3634 3470-82 10.1002/sim.3634 19536744

# Response

**Published:** 2022-03-16

**Authors:** Arathi Rao

**Affiliations:** Manipal College of Dental Sciences, Mangalore, Manipal Academy of Higher Education, Manipal, India

- *Abstract: It has few typos such as - First line in Methods - replace "a randomized controlled trial" with " as a randomized controlled trial" or " A double blinded, split mouth randomized controlled trial was carried out  *
***   *    **
- **Response: **Correction done.
- *Title: Can be altered to - Comparison of Clinical Effectiveness of Conventional and Self-etch Sealant - A split mouth Randomized Controlled Trial*
- **Response: **Changes done.
- *Introduction: ****The need for this research could have been emphasized in the introduction section. *
- **Response:** It's mentioned as follows: Considering the compliance of the young child, a sealant that provides good retention and having a short procedure of application would be ideal. ^6 ^Self-etching sealants are products that have a one-step application with fluoride releasing properties. They can thus prove to be beneficial compared to the other resin sealants especially in pediatric practices. ^2 ^There have been many *in-vitro* evaluation studies ^6,7 ^conducted comparing the self-etch sealant with conventional acid etch sealants. The present *in-vivo* study was thus designed with the focus to clinically evaluate the sealing ability and retention of the self-etching sealant compared to the conventional resin sealant.
- *The heading Objective and Hypothesis to be removed and the last paragraph to be merged with Introduction.*
- **Response: **Changes done.
- *Methods: ****Design cannot be both - Parallel and Split mouth. *
- **Response: **Corrections done.
- *Randomization - Too many repetitions.*
- **Response: **Repetitions removed.
- *Was any allocation concealment was done? *
- **Response: **Since the children are selected based on inclusion and exclusion criteria and both the test and control groups are in the given child- the risk of selection bias is minimum. Also the coin was tossed just before the procedure.
- *Page 4 - Intervention - the statements ‘ The trial commenced from March 2019 to April 2020 and all the follow-up examinations were completed by 15th April 2021. Both the study materials were purchased by the investigators and were used within their labeled shelf life’ cannot be the intervention, please check and rephrase.*
- **Response: **Corrections done.
- *Page 6, third line -Please mention after how many months in the statement - ‘Complete retention of sealants was observed in 34.5% of group 1 and 22.9% of group 2 samples, whereas complete loss of sealants was seen in 11.4% of group 1 and 20% of the group 2 samples’.*
- **Response: **Changes done and sentence reframed.
- *Discussion: It needs to be re-structured.*
- **Response: **Changes done.
- *The results need to be compared with previous studies.*
- **Response: **Similar studies were included as reference 13 and 14 but uses self etch adhesive liners and not self etch sealants. Ref 8 uses self etch sealants but compared microleakage which was beyond the scope of the present study. Ref No 6 is the only in vitro study done comparing the study materials and evaluation criteria.
- *Please mention limitations about carrying out toss coin randomization.*
- **Response: **Changes made.
- *Please highlight the study limitations of using a split mouth study design - First, the split-mouth design may lead to biased treatment efficacy estimates due to carry-across effects which (most likely) will be low or absent. However, there is even no statistical test that can detect this carry-across effect. Second, bias in recruitment of patients because of the need for symmetrical disease patterns among all segments of the dentition that are randomized and this limits the external validity of the results. Please refer to Lesaffre et al. (2009 ^1^ ) for more details.*
- **Response: **Limitations added.
- *Fluoride releasing ability of Preventseal wasn’t assessed in the present study hence in my opinion it shouldn’t be included here. *
- **Response: **Changes done. 
- *There is room for improvement. Professional language edit of the entire manuscript can be helpful. Either American or British English to be used throughout.*
- **Response: **Language re checked and changes made.

# Peer review: Reviewer response for version 1

**DOI:** 10.5256/f1000research.121099.r126007

**Published:** 2022-03-07

**Authors:** Parajeeta Dikshit

**Affiliations:** 1 Department of Pedodontics, Kantipur Dental College, Basundhara, Nepal

Based on my expertise I have found the format of the article is well designed. The abstract is concise and has given the needed gist of the article. The study design is transparent with no chances of biases. The authors have discussed and compared the results with other researchers adequately. The article can be indexed as it is.

Is the work clearly and accurately presented and does it cite the current literature?

Yes

If applicable, is the statistical analysis and its interpretation appropriate?

I cannot comment. A qualified statistician is required.

Are all the source data underlying the results available to ensure full reproducibility?

Yes

Is the study design appropriate and is the work technically sound?

Yes

Are the conclusions drawn adequately supported by the results?

Yes

Are sufficient details of methods and analysis provided to allow replication by others?

Yes

Reviewer Expertise:

Pediatric Dentistry

I confirm that I have read this submission and believe that I have an appropriate level of expertise to confirm that it is of an acceptable scientific standard.
